# Machine learning and public health policy evaluation: research dynamics and prospects for challenges

**DOI:** 10.3389/fpubh.2025.1502599

**Published:** 2025-01-30

**Authors:** Zhengyin Li, Hui Zhou, Zhen Xu, Qingyang Ma

**Affiliations:** ^1^Institute of Agricultural Economics and Development, Chinese Academy of Agricultural Sciences, Beijing, China; ^2^School of Law, University of Chinese Academy of Social Sciences, Beijing, China

**Keywords:** public health policy evaluation, machine learning, big data, DID, RDD, SCM

## Abstract

**Background:**

Public health policy evaluation is crucial for improving health outcomes, optimizing healthcare resource allocation, and ensuring fairness and transparency in decision-making. With the rise of big data, traditional evaluation methods face new challenges, requiring innovative approaches.

**Methods:**

This article reviews the principles, scope, and limitations of traditional public health policy evaluation methods and explores the application of machine learning in evaluating public health policies. It analyzes the specific steps for applying machine learning and provides practical examples. The challenges discussed include model interpretability, data bias, the continuation of historical health inequities, and data privacy concerns, while proposing ways to better apply machine learning in the context of big data.

**Results:**

Machine learning techniques hold promise in overcoming some limitations of traditional methods, offering more precise evaluations of public health policies. However, challenges such as lack of model interpretability, the perpetuation of health inequities, data bias, and privacy concerns remain significant.

**Discussion:**

To address these challenges, the article suggests integrating data-driven and theory-driven approaches to improve model interpretability, developing multi-level data strategies to reduce bias and mitigate health inequities, ensuring data privacy through technical safeguards and legal frameworks, and employing validation and benchmarking strategies to enhance model robustness and reproducibility.

## 1 Introduction

Policy assessment constitutes a vital component of the national governance framework (1). A scientific and accurate assessment of policy impacts is essential for the modernization of the national governance system and its capacity (2). Traditional policy assessment predominantly utilizes data from sources like official statistical yearbooks, publicly accessible questionnaire databases, and field surveys. The main objective of policy assessment is to empirically validate economic theories (3). In contrast, public health policy evaluations often require integrating various data sources, such as epidemiological, healthcare, and socioeconomic data. The diversity and complexity of data involved in public health assessments are more pronounced compared to other policy evaluations. Additionally, due to the sensitive nature of health data, these evaluations must carefully address data privacy and ethical concerns to ensure individual rights and privacy are protected during data usage. With the advent of the digital economy, driven by the rise of the Internet, cloud computing, and artificial intelligence, many biological data and medical records have been digitized. This has introduced new types of data, such as text, image, and audio data, which are typically unstructured, high-dimensional, low in information density, and large in scale, exceeding the capabilities of traditional public health policy evaluation methods (4). Introducing machine learning methods is essential for effectively exploring and managing these data.

Machine learning is an emerging technology that integrates knowledge from diverse fields such as computer science, engineering, and statistics. It is widely utilized in areas including science, technology, and medicine, and is increasingly attracting interest from researchers in the social sciences (5). The advent of machine learning offers a novel approach to public health policy evaluation. By leveraging advanced algorithms and significant computational power, machine learning can extract critical insights from large datasets and develop more precise predictive models, thereby providing more reliable references for public health policy formulation. Currently, machine learning has become widely prevalent in social science research, leading to numerous review articles focusing on machine learning and causal relationships (6). For example, Grimmer (7), Varian (8), Guo et al. (9). Provide brief introductions to the fundamental concepts and logic of causal identification, explore the intersection of machine learning with causal identification, and discuss the potential of integrating machine learning models into causal identification processes. Mullainathan and Spiess (10), Guo and Tao (11) underscore the critical role of machine learning in causal identification within social sciences, while also addressing the novel challenges it introduces. The existing literature primarily explores the relationship between machine learning and causal identification and emphasizes the significance of incorporating machine learning methods for causal identification within the context of big data. This article, however, emphasizes the value of machine learning in public health policy effect evaluation. Related literature includes research by Shen et al. (12), who compare traditional policy evaluation methods such as the difference-in-differences method, synthetic control method, and panel data method with machine learning approaches, offering empirical researchers a guide for method selection. Nevertheless, this article differs significantly: Shen et al. (13) primarily introduce traditional and machine learning-based policy evaluation methods, conducting a detailed comparison for methodological selection. In contrast, this article delves into the limitations of traditional public health policy evaluation methods within the context of big data and provides a comprehensive exploration of expanding public health policy evaluation boundaries using machine learning methods. Unlike Shen et al.’s straightforward comparison, this article presents a more thorough and detailed examination of machine learning’s application in public health policy evaluation.

This study aims to address the following key question: How can machine learning techniques improve the evaluation of public health policies in the era of big data, and what challenges and solutions are associated with their application? Hence, the primary contributions of this paper are twofold: first, it outlines the challenges encountered by traditional public health policy effect assessment methods in the context of big data and highlights the advantages and significant value of machine learning in this field. It also offers new insights for scholars seeking to expand the applicability of public health policy effect assessment. Second, the article addresses the limitations of machine learning in public health policy effect assessment and suggests potential directions and efforts for the further development of public health policy assessment and machine learning. The structure of the remaining sections in this article is as follows: Section 2 introduces traditional methods of public health policy impact assessment and examines their challenges in the context of big data. Section 3 highlights the advantages and substantial value of machine learning in public health policy impact assessment within the realm of big data. Section 4 discusses the limitations of machine learning in public health policy impact assessment and proposes future directions. Finally, Section 5 provides a comprehensive summary of the entire paper.

## 2 Traditional methods of public health policy impact assessment and their existing issues

### 2.1 Traditional methods of public health policy assessment

The field of econometrics has developed several traditional methods for quantitatively analyzing public health policy effectiveness. Key among these are the difference-in-differences (DID), synthetic control method (SCM), and regression discontinuity design (RDD), which have gained prominence in academia and are extensively used across various public health policy domains. This section examines the principles, applicability, specific model configurations, and potential challenges related to these conventional public health policy evaluation methods.

#### 2.1.1 Difference-in-difference (DID)

The difference-in-differences (DID) method, originally introduced by Card et al. in their study of minimum wage policy, has been widely adapted for use in public health policy evaluation (14). This method assesses the effects of public health interventions by comparing the differences in outcomes between treatment and control groups before and after a policy is implemented. The first step involves calculating the difference in health outcomes for individuals before and after the policy intervention, which helps control for individual fixed effects (unchanging characteristics specific to each individual). The second step takes a secondary difference, focusing on time-based differentiation across the treatment and control groups, which aims to eliminate time fixed effects (common factors that remain consistent over time). This approach is particularly valuable in public health when policies are tested in certain regions or populations before being rolled out more broadly. It provides a robust way to assess the impact of interventions by controlling for both individual and time-specific effects. However, public health policies implemented simultaneously across all regions may not be well-suited for the DID method. Generally, the model specification for DID in public health policy evaluation follows this structure:


(1) $$\begin{array}{l} Y_{\mathrm{it}}=\beta_{0}+\beta_{1}{\mathrm{Treat}}_{i}+\beta_{2}{\mathrm{Post}}_{t}+\beta_{3}{\mathrm{Treat}}_{i}{\mathrm{Post}}_{t}\\ +\overset{J}{\underset{j=1}{\sum }}\theta_{j}Z_{ijt}+\mu_{i}+\delta_{t}+\varepsilon_{\mathrm{it}} \end{array}$$

In Equation (1), subscript i denotes the sample and t denotes time. $Y_{\mathrm{it}}$ represents the dependent variable, indicating the outcome for sample i at time t . ${\mathrm{Treat}}_{i}$ is a dummy variable indicating whether the sample is subject to the public health policy intervention, assigned 1 if affected by the policy, otherwise 0. ${\mathrm{Post}}_{t}$ is a dummy variable for the policy implementation time point, 0 before and 1 after implementation. The term ${\mathrm{Treat}}_{i}{\mathrm{Post}}_{t}$ represents the interaction term in the difference-in-differences method, used to identify policy effects. If $\beta_{3}>0$ , the policy increased $Y_{\mathrm{it}}$ ; if $\beta_{3}<0$ , it decreased $Y_{\mathrm{it}}$ . $Z_{ijt}$ is the matrix of control variables, including factors affecting the dependent variable. $\mu_{i}$ and $\delta_{t}$ are individual and time fixed effects, respectively. $\varepsilon_{\mathrm{it}}$ represents the random disturbance term.

**Figure 1:**
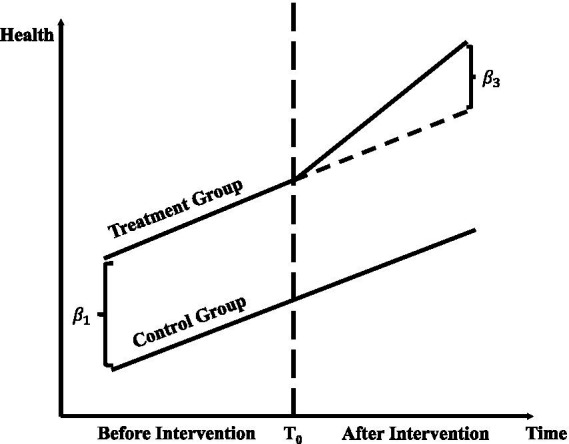
Difference-in-differences (DID) method.

Figure 1 demonstrates the application of the Difference-in-Differences (DID) methodology in evaluating public health policy interventions. It highlights changes in outcome variables, such as health expenditures and mortality rates, between treatment and control groups before and after the intervention. The observed parallel trends in the pre-intervention period confirm the validity of the DID approach, while the divergence in outcomes post-intervention quantifies the policy’s causal impact. By incorporating machine learning into this framework, the study enhances policy evaluation by capturing complex, non-linear relationships and systemic factors, such as hospital ownership. This integration establishes a robust empirical foundation for analyzing public health outcomes and assessing policy effects.

The double-difference method may encounter challenges related to the parallel trend assumption and selection bias in evaluating public health policy effects. This method relies on the parallel trend assumption, which posits that the health trends of the treatment and control groups are parallel before and after the implementation of public health policies. However, fulfilling this assumption is often difficult in real-world evaluations, particularly when external health interventions or uncontrollable factors, such as epidemics, influence the policy implementation. If the parallel trend assumption is violated, it can result in significant bias in estimating policy effects, thereby compromising the accuracy and reliability of public health assessments. Additionally, the double-difference method is susceptible to selection bias. If, following the implementation of a public health policy, the differences between the treatment and control groups arise not only from the policy effects but are also influenced by other health-related factors (e.g., health infrastructure, access to medical services), the double-difference method may not accurately assess the actual effects of the policy, leading to distorted evaluation results.

In the context of big data and machine learning, the Difference-in-Differences (DID) method can be improved by incorporating machine learning algorithms, such as propensity score matching (PSM) or random forests, to construct balanced treatment and control groups that more effectively satisfy the parallel trends assumption. For instance, machine learning models can analyze high-dimensional datasets to identify key covariates influencing treatment assignment, thereby enhancing the robustness of DID analysis. Additionally, DID can be adapted to process unstructured data, such as social media health posts or electronic medical records, by employing natural language processing (NLP) or image recognition techniques, expanding its applicability in public health contexts.

#### 2.1.2 Synthetic control method (SCM)

The synthetic control method (SCM) extends the double-difference method by serving as a counterfactual for the treatment group. In evaluating the effects of public health policies, it is often challenging to identify an optimal control group that closely matches the treatment group in all relevant aspects. SCM, based on the counterfactual estimation framework, offers a viable solution. The core idea is that, although it is difficult to find ideal control samples identical to the subjects undergoing policy intervention, a control group can be constructed by linearly combining weights from a pool of potential control groups. This synthetic control group can mirror the characteristics of the treatment group prior to policy implementation, ensuring that the predictor variables align with those of the treated sample. Consequently, it allows for a comparison of changes in health variables between the “real treatment group” and the “synthetic control group” before and after policy implementation, ultimately revealing the net effect of the public health policy (15). This approach is particularly suitable for public health policies with few pilot programs, as they may be tested in only one or two regions, making it difficult to find exact matches elsewhere. Thus, constructing a synthetic counterfactual reference group through an appropriate linear combination of non-pilot districts becomes essential. Typically, the synthetic control method follows four key steps:

Firstly, the model specification assumes an experimental group where the public health policy is implemented, and multiple control groups where the public health policy is not implemented. For each sample i and time t, the model is represented as follows:


(2) $$Y_{\mathrm{it}}=\beta_{0}+\beta_{1}{\mathrm{Treat}}_{\mathrm{it}}+\overset{J}{\underset{j=1}{\sum }}\theta_{j}Z_{ijt}+\mu_{i}+\delta_{t}+\varepsilon_{\mathrm{it}}$$

In Equation (2), Subscript i denotes the sample and t denotes time. $Y_{\mathrm{it}}$ is the dependent variable, representing the outcome for sample i at time t . ${\mathrm{Treat}}_{\mathrm{it}}$ is a dummy variable indicating public health policy implementation, assigned 1 if the policy is implemented for sample i , and 0 otherwise. $Z_{ijt}$ represents the matrix of control variables, including factors affecting the dependent variable. $\mu_{i}$ and $\delta_{t}$ denote individual and time fixed effects, respectively. $\varepsilon_{\mathrm{it}}$ represents the random disturbance term.

Secondly, the synthetic control group is constructed for each sample i where the public health policy is not implemented, by weighting and averaging observed data from other control group samples.


(3) $$\hat{Y_{\mathrm{it}}^{s}}=\overset{J}{\underset{j=1}{\sum }}W_{ij}Y_{jt}$$

In Equation (3), $\hat{Y_{\mathrm{it}}^{s}}$ denotes the predicted value of the synthetic control group; j represents the number of reference samples used to construct the synthetic control group; $W_{ij}$ represents the weights computed for each reference sample j .

Thirdly, synthetic weights are determined by minimizing the differences observed between the experimental group and the synthetic control group before public health policy implementation.


(4) $$\min\overset{T_{1}}{\underset{t=T_{0}}{\sum }}{\left(Y_{\mathrm{it}}-\overset{J}{\underset{j=1}{\sum }}W_{ij}Y_{jt}\right)}^{2}$$

In Equation (4), $T_{0}$ and $T_{1}$ denote the time points before and after public health policy implementation, respectively.

Fourthly, as shown in Equation (5), public health policy effects are estimated by comparing the observed values of the experimental group with the predicted values of the synthetic control group.


(5) $$\mathrm{Policy Effects}=Y_{\mathrm{it}}-\hat{Y_{\mathrm{it}}^{s}}$$

**Figure 2:**
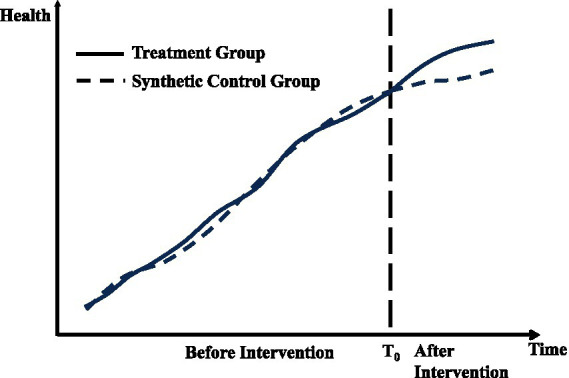
Synthetic control method.

Figure 2 demonstrates the application of the synthetic control method (SCM) in the context of public policy evaluation. The x-axis represents the time period, while the y-axis indicates the outcome variable of health. The solid line depicts the actual observations for the treated unit, whereas the dashed line represents the estimated counterfactual outcomes generated by the synthetic control group. Before the intervention, marked by a vertical line, the treated unit and the synthetic control group exhibit a similar trajectory, confirming that the SCM effectively approximates the pre-intervention trend of the treated unit. After the intervention, a noticeable divergence between the two lines emerges, reflecting the treatment effect. This gap quantifies the impact of the intervention on the treated unit, assuming the synthetic control group serves as a valid counterfactual.

The synthetic control method may encounter selection bias in public health policy evaluation due to synthetic portfolio construction. This method necessitates the creation of a synthetic portfolio to simulate the post-policy implementation scenario for comparison with the actual treatment group. However, the selection process may be influenced by the researcher’s subjective preferences and methodological choices, leading to biased outcomes. For instance, a researcher might favor synthetic combinations that demonstrate more favorable effects post-implementation or might rely on specific techniques or models while overlooking alternative methods that could be more suitable. Such subjective and technical decisions can yield synthetic combinations that do not accurately reflect the actual context and compromise the validity and reliability of the policy assessment. Furthermore, public health policies typically target unique intervention objectives and specific populations, making it challenging to identify an appropriate control group. This limitation further diminishes the objectivity and accuracy of the evaluation results. Consequently, the validity and applicability of the synthetic control method in public health policy evaluation are subject to significant constraints.

In the era of big data, machine learning algorithms, such as k-means clustering and neural networks, can automate the selection of control units, thereby minimizing subjectivity in the construction of synthetic portfolios. These algorithms detect patterns and similarities in large-scale datasets, facilitating the creation of more representative synthetic control groups. For instance, when evaluating a public health policy targeting specific regions, unsupervised learning techniques can group similar regions based on health indicators, socioeconomic variables, and other high-dimensional features, thereby enhancing the accuracy and credibility of synthetic control method (SCM) applications in public health.

#### 2.1.3 Regression discontinuity design (RDD)

The regression discontinuity design (RDD) was initially proposed by Thistlewaite and Campbell (16), but it gained widespread attention and application in 2001 following Hahn et al.’s formal proof (17). Currently, RDD is extensively used by social science researchers to evaluate public health policies in non-experimental settings (18). The core idea is that when individual characteristics of a subgroup cross a specific policy threshold, the policy induces a discontinuity in the outcome variable. Near the discontinuous policy threshold, groups on both sides form “comparable” experimental and control groups. Given the similarity of these groups, any disparities in the outcome variable are solely attributable to the policy intervention. The breakpoint regression model is ideal for “one-size-fits-all” policies that necessitate explicit policy thresholds, where entities must surpass (or fall below) these thresholds to fall under the policy’s purview. Typically, breakpoint regression models are structured as follows:


(6) $$\begin{array}{l} Y_{\mathrm{it}}=\gamma_{0}+\gamma_{1}\left(X_{\mathrm{it}}-c\right)+\gamma_{2}D_{\mathrm{it}}+\gamma_{3}\left(X_{\mathrm{it}}-c\right)•D_{\mathrm{it}}\\ +\overset{J}{\underset{j=1}{\sum }}\theta_{j}Z_{ijt}+\mu_{i}+\delta_{t}+\varepsilon_{\mathrm{it}} \end{array}$$

In Equation (6), subscript i denotes the sample and t denotes time. $Y_{\mathrm{it}}$ is the dependent variable indicating the outcome for sample i at time t . $X_{\mathrm{it}}$ represents the continuous variable related to the public health policy observed for individual i at time t . c denotes the public health policy threshold location. $D_{\mathrm{it}}$ is an indicator variable, equaling 1 when $X_{\mathrm{it}}$ exceeds c and 0 otherwise. $\gamma_{1}$ is the coefficient of $X_{\mathrm{it}}$ , reflecting the slope near the public health policy threshold. $\gamma_{2}$ is the coefficient of $D_{\mathrm{it}}$ , indicating the public health policy effect on the outcome variable. $\gamma_{3}$ represents the coefficient of the interaction term capturing the discontinuity effect due to the public health policy. $Z_{ijt}$ is a matrix of control variables affecting the dependent variable. $\mu_{i}$ and $\delta_{t}$ denote individual and time fixed effects, respectively. $\varepsilon_{\mathrm{it}}$ represents the random disturbance term.

**Figure 3:**
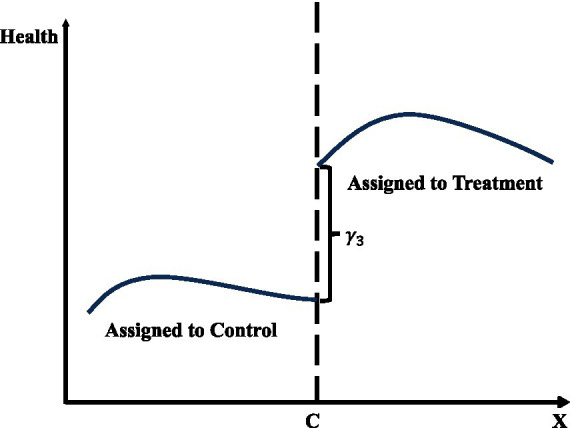
Regression discontinuity design (RDD).

Figure 3 demonstrates the central concept of regression discontinuity design (RDD) by depicting the relationship between the running variable (x-axis) and the outcome variable (y-axis). The graph features two fitted lines, each representing observations on either side of the threshold, marked by a vertical dashed line. The left segment corresponds to the control group, while the right segment represents the treatment group. At the threshold, the discontinuity, or “jump,” in the outcome variable indicates the causal effect of the treatment, provided the model’s validity conditions are satisfied. The curvature in the fitted lines reflects non-linear relationships in the data, which are addressed through the chosen modeling strategy, such as higher-order polynomials or machine learning algorithms.

Regression discontinuity design may suffer from breakpoint selection bias in public health policy evaluation. The core of this model relies on a predetermined breakpoint to distinguish between treatment and control groups. However, the selection of breakpoints can be influenced by the researcher’s subjective preferences or external factors, leading to inappropriate or inaccurate choices. In public health policy, breakpoints may be unclear and challenging to define. If a researcher arbitrarily selects a breakpoint without adequate justification, it can result in significant bias in the assessment results, thereby affecting the accurate evaluation and interpretation of policy effects. For instance, a researcher might search for the most optimal breakpoints in the data or choose different breakpoints before and after the implementation of the policy to obtain more favorable results. Such subjectivity and flexibility in breakpoint selection may compromise the objectivity and credibility of the assessment outcomes. Additionally, regression discontinuity design relies on the uniform distribution of the sample on both sides of the cutoff point. If the sample size is insufficient or if there is systematic selection bias on either side of the cutoff, the reliability of the assessment results may be further weakened.

In the context of big data and machine learning, Regression Discontinuity Design (RDD) can be enhanced by leveraging algorithms such as gradient boosting or support vector machines to identify breakpoints more objectively. These methods analyze high-dimensional datasets to detect natural thresholds or discontinuities in the data, thereby reducing reliance on arbitrary or subjective breakpoint selection. Additionally, RDD can be extended to fuzzy designs using machine learning techniques to predict treatment probabilities when treatment assignment is not perfectly determined by the threshold. For example, in a vaccination program study, machine learning models can estimate individual-level probabilities of receiving treatment near the threshold, thereby enhancing the robustness of RDD analysis.

### 2.2 The problems of traditional public health policy assessment methods in the context of big data

The information technology revolution, driven by the Internet, cloud computing, and artificial intelligence, has facilitated the widespread adoption of digital technology in public health. Many aspects of human health behavior and medical activities are now digitally recorded, resulting in vast amounts of new data that encompass dynamic information on individual health status and healthcare service usage. These data are interrelated in complex ways (19), and traditional public health policy evaluation methods are often insufficient for processing and analyzing such complex datasets (20).

#### 2.2.1 Unstructured data challenges and huge volume difficulties

Unstructured data in the era of big data presents new challenges for public health policy evaluation. Traditional assessments primarily rely on structured data, such as health statistics yearbooks, medical record databases, or field surveys, which have clear formats and fixed fields, making them easier to analyze and model. However, with the rapid advancement of computer information technologies like the Internet, cloud computing, and artificial intelligence, unprecedented and diverse data types—such as text, images, and audio—are now available, posing challenges for traditional public health evaluations (21). Big data is characterized by unstructured formats, high dimensionality, and low information density (22), creating complexity for traditional evaluation methods. Unstructured data, including electronic medical records, health monitoring data, and social media health information, differs from structured data in its flexibility and diversity. Traditional methods are ill-equipped to extract and analyze such data. High dimensionality involves numerous features and attributes, such as individual health behaviors and environmental factors, exceeding the capacity of traditional tools. Moreover, big data’s low information density requires sophisticated techniques to extract meaningful insights, making manual analysis insufficient for processing high-noise data. Additionally, the sheer volume of big data poses significant challenges in terms of storage, transmission, and processing (23). Public health policy evaluation often necessitates the integration of data from multiple domains, such as medical, social, and environmental sources. This may involve merging multiple large databases, which traditional computing methods handle inefficiently. Thus, advanced technologies like parallel computing and distributed storage are essential for efficiently processing large-scale data and ensuring the accuracy and timeliness of public health policy evaluations.

#### 2.2.2 Model specification issues

##### 2.2.2.1 Misspecification issue

Quantitative empirical research on public health policy assessment can be categorized into two types: theory-driven and data-driven approaches. Theory-driven research, which traditionally dominates this field, constructs low-dimensional parametric models based on public health theories. However, these theories are often derived from mathematical models built on numerous assumptions, aiming to simplify and abstract complex health systems. This method focuses on identifying causal relationships between key health indicators and policy interventions to reveal the intrinsic effects of policies on health outcomes. The advantages of the theory-driven approach lie in its model simplicity, ease of understanding, and straightforward result interpretation. In macroeconomics, the Lucas Critique illustrates how dynamic, theory-driven models integrate rational expectations to improve predictions of policy impacts, thereby avoiding the limitations of purely data-driven models. Similarly, in psychology, cognitive behavioral therapy (CBT) utilizes behavioral theories to explain the interaction between cognitive distortions and behavioral outcomes, offering a structured and interpretable framework for clinical interventions. These examples highlight the strength of theory-driven approaches in promoting deeper understanding and more effective application of models. However, its limitations stem from reliance on *a priori* knowledge and theoretical assumptions. Model construction depends on the researcher’s prior understanding of the health problem and domain knowledge, making it difficult to handle complex big data. This approach may overlook the intricate relationships and nonlinear patterns present in large datasets. Consequently, theory-driven models may struggle to capture deeper insights in high-dimensional health data, leading to potential model misspecification and impeding accurate public health policy evaluation (4).

##### 2.2.2.2 Multicollinearity issue

In the era of big data, public health datasets contain numerous variables, resulting in high-dimensional data. This increase in dimensionality amplifies the correlation between variables, heightening the risk of multicollinearity, which threatens both the accuracy and interpretability of models (24). Multicollinearity arises from the interrelationships between health-related variables within large datasets. Big data encompasses vast and complex information across multiple domains, including individual health status, socioeconomic factors, and environmental conditions. Traditional methods for evaluating public health policies struggle to identify correlations among variables within such large-scale data, potentially leading to multicollinearity. This complicates the accurate estimation of independent contributions from variables in traditional models, resulting in unstable and unreliable coefficient estimates, which can even lead to incorrect evaluations of public health policies, ultimately undermining the scientific rigor and effectiveness of policy decisions.

##### 2.2.2.3 Overfitting issue

Traditional models for assessing the effects of public health policies typically involve low-dimensional explanatory variables and unknown parameters. Researchers primarily focus on consistently estimating these unknown parameters and assessing the statistical significance of their estimates using the t-statistic or *p*-value, thus inferring their actual impact on health outcomes. For instance, when the t-statistic exceeds its critical value, researchers may reject the null hypothesis (commonly the hypothesis that the parameter equals zero) in favor of the alternative hypothesis, concluding that the parameter estimate is statistically significant and has a meaningful impact on public health. However, in the context of big data, large sample sizes often result in parameter estimates closely aligning with true values or their probability limits, accompanied by small standard errors. Consequently, even when true parameter values are near zero and their actual impact on health outcomes is minimal, the t-values may still achieve statistical significance, raising concerns about overfitting. Therefore, while the distinction between statistical significance and actual significance of public health effects is less critical with smaller data volumes, it becomes essential to differentiate between the two in big data contexts (4). Otherwise, a parameter deemed statistically significant may not possess sufficient actual impact on public health to support substantive policy conclusions.

## 3 The advantages of machine learning in public health policy impact assessment

Big data, characterized by unstructured formats, high dimensionality, low information density, and large volumes, poses significant challenges for traditional public health policy evaluation methods. Issues such as model misspecification, multicollinearity, and overfitting are increasingly prominent in this context. Machine learning offers a solution to these challenges by flexibly handling large-scale, high-dimensional health data, uncovering hidden associations, identifying nonlinear relationships, and reducing dependence on *a priori* knowledge through automatic feature extraction.

### 3.1 Addressing challenges of unstructured data and massive volume

In the era of big data, machine learning offers a flexible and adaptive tool to address the challenges posed by unstructured data in public health policy evaluation. Machine learning is particularly effective in handling unstructured, high-dimensional, and low-information-density data. For unstructured health data, such as text, images, and audio from sources like consultation records and medical imaging, machine learning—particularly deep learning models—leverages its capability to process unstructured information. These models comprehensively analyze textual health content, recognize patterns in medical images, and interpret audio data, thereby extracting diverse health-related insights and aiding in the deeper analysis of public health policy effects (25). Machine learning algorithms also manage high-dimensional health data by utilizing techniques like feature selection and dimensionality reduction, enabling automatic identification of key features linked to policy effects and alleviating the burden of dimensionality on traditional methods (26). Additionally, machine learning excels at extracting valuable information from noisy health data, where traditional methods struggle, improving both the efficiency and accuracy of data analysis. Furthermore, machine learning addresses the challenges of storing, transmitting, and processing large-scale health data using advanced technologies such as parallel computing and distributed storage. Parallel computing allows for the simultaneous execution of multiple tasks, boosting computational efficiency, while distributed storage enables the decentralized management of large datasets across multiple nodes, enhancing data access speed and overall processing capacity. These technologies make machine learning models more efficient in handling large-scale health data, meeting the real-time and precision demands of public health policy evaluation. In summary, machine learning algorithms provide a comprehensive approach to extracting and analyzing big health data, offering policymakers more accurate and reliable assessments of public health policy effects.

### 3.2 Addressing issues in traditional public health policy evaluation models

#### 3.2.1 Data-driven solutions to address mis-specification issues

Machine learning mitigates the risk of model mis-specification in public health policy evaluation through a data-driven approach. Traditional modeling often relies on specific theoretical frameworks that may simplify actual conditions. As a result, it typically employs linear models, such as linear regression, which assume a linear relationship between variables. However, these assumptions may be overly simplistic and fail to accurately capture the potential nonlinearities and interactions among variables when applied to complex public health phenomena in the context of big data and heterogeneity. Furthermore, manually incorporating these relationships can lead to model mis-specification, biasing the estimated parameters. In contrast, machine learning algorithms, such as decision trees and support vector machines, effectively capture nonlinear relationships without the risk of biased estimates resulting from model mis-specification (27, 28). Additionally, ensemble algorithms in machine learning, such as Super Learner, Bayesian Stacked Regression Trees, and Deep Learning, are designed to optimize model specification. These algorithms utilize foundational models trained independently on the same samples, leveraging relatively weak learning models and subsequently integrating their results for improved generalization. By evaluating the strengths of each model, ensemble algorithms aim to identify the optimal model, thereby significantly reducing the risk of mis-specification. This approach provides researchers with more flexible and adaptable tools for understanding and interpreting patterns and associations in large-scale health data, ultimately enhancing the accuracy and reliability of public health policy effect assessments.

#### 3.2.2 Dimensionality reduction of high-dimensional models resolves multicollinearity issues

Dimensionality reduction in high-dimensional models is an effective strategy for addressing multicollinearity issues in the assessment of public health policy effects. Reducing the number of variables in the model decreases correlations between them, thereby improving the stability and interpretability of the model. The primary objective of dimensionality reduction in high-dimensional models is to preserve essential information while eliminating redundant dimensions, aiming for a more concise and interpretable model. Common dimensionality reduction methods include Principal Component Analysis (PCA), Singular Value Decomposition (SVD), and other techniques. These methods transform the original explanatory variables through linear transformations to produce a new set of variables known as principal components. These principal components are linear combinations of the original variables designed to preserve as much variance from the original data as possible. Selecting a subset of principal components enables dimensionality reduction of the original high-dimensional data, thereby reducing the number of explanatory variables in the model and mitigating multicollinearity issues. Multicollinearity arises from high correlations between variables, and principal components are typically constructed by identifying directions of maximum variance, making them orthogonal to each other. This orthogonality implies that correlations between variables are significantly reduced in the space of principal components, thereby mitigating the impact of multicollinearity (29). Selecting principal components allows us to focus on the most representative directions in the public health data, condensing the information of the original data into fewer dimensions. This approach captures the essential characteristics of the data more effectively. However, it is crucial to carefully select the number of principal components to retain during dimensionality reduction. Choosing too few components may lead to loss of important information, while selecting too many may not effectively reduce dimensionality. A common method is to determine the number of principal components based on the cumulative contribution of explained variance (30). Typically, the number of principal components chosen achieves a cumulative contribution rate above a certain threshold, ensuring sufficient information retention while reducing dimensionality.

#### 3.2.3 Regularization addresses overfitting problems

Regularization mitigates model complexity by introducing a penalty term into the model’s loss function of the public health policy effects assessment model, thereby preventing overfitting to the training data and enhancing its generalization ability on unseen data. The central concept of regularization is to balance the model’s fit and complexity to avoid overfitting. The most commonly used regularization methods are L1 regularization (Lasso) and L2 regularization (Ridge) (31). L1 regularization sparsifies the model by incorporating the L1 norm of the parameter vectors into the loss function. This approach drives some model coefficients to zero, thereby reducing the impact of less important features. Conversely, L2 regularization encourages smaller coefficient values by adding the L2 norm of the parameter vectors to the loss function. This effectively controls the model’s weight size and mitigates overfitting to noise. Additionally, regularization is manifested through the penalty term in the model. This term modifies the model’s loss function to ensure that the model not only fits the training data well but also keeps its parameters within a reasonable range. Consequently, the model emphasizes capturing the essential patterns in the public health data while minimizing overfitting to noise. The strength of regularization is controlled by a tuning parameter, which, when adjusted, helps strike a balance between model complexity and fit.

## 4 Applying machine learning for public health policy evaluation

### 4.1 Application steps

Machine learning has emerged as a powerful tool for evaluating policy effectiveness in the context of big data, addressing limitations associated with traditional methods. Using text data as an illustrative example, this article explores the application of machine learning in policy evaluation, focusing on critical steps such as data preparation and cleaning, model selection, training and tuning, model interpretation, and result analysis. The objective is to assist researchers in effectively utilizing this advanced methodology.

#### 4.1.1 Data preparation and cleaning

The process of preparing and cleaning data for machine learning begins with text data mining. Natural language processing (NLP) techniques, such as word vectors and bag-of-words models, are employed to analyze text structure and semantics, extracting key information to generate meaningful features for subsequent processing. The next step addresses missing and outlier data through techniques like random forests and k-nearest neighbors for predicting and imputing missing values. Outlier detection methods, including isolation forests and One-Class SVM, are applied to identify and manage anomalies, thereby enhancing data quality. Data smoothing and transformation follow, utilizing approaches like moving averages and exponential smoothing to stabilize time series data, reduce noise, and improve interpretability. Subsequently, feature engineering techniques, such as principal component analysis (PCA) and feature selection methods (e.g., variance thresholding), are employed to extract critical features, optimizing model inputs and improving generalization. The final step standardizes and normalizes data using techniques such as Z-score standardization and MinMax normalization, ensuring consistent feature scales to mitigate bias and provide stable inputs for model training.

#### 4.1.2 Model selection, training and tuning

After data processing and cleaning, the next critical step is model selection, training, and tuning, which significantly impacts model performance. The initial phase involves selecting candidate models, such as decision trees, support vector machines, random forests, linear regression, logistic regression, k-nearest neighbors, naïve Bayes, and neural networks, to encompass diverse modeling approaches. Unlike traditional methods, this process integrates data-driven strategies with domain expertise and policy theories, fostering the development of interpretable and credible models. Model training and hyperparameter tuning then proceed, using cross-validation techniques like k-fold cross-validation to partition the dataset into training and validation sets. Techniques such as grid search, random search, Bayesian optimization, genetic algorithms, gradient boosting, and automated machine learning (AutoML) are employed to identify optimal hyperparameter configurations, guided by policy considerations. These configurations are selected based on validation set performance. Subsequently, model comparison and selection evaluate metrics like accuracy, precision, F1 score, AUC-ROC curves, and confusion matrices to identify the best-performing model. This evaluation integrates domain knowledge, ensuring alignment with broader policy objectives. Finally, ensemble learning techniques, including voting classifiers, stacked models, random forests, gradient boosting trees, and deep learning ensembles, are employed to enhance generalization capabilities. The finalized model is validated using an independent test set to ensure robust performance on unseen data. The models available for each step are shown in Table 1.

**Table 1:** Models available for model selection, training and tuning.

Step	Available models
Initial model selection	Decision trees, support vector machines, random forests, linear regression, logistic regression, k-nearest neighbors, naïve Bayes, neural networks, etc.
Model training and tuning	Grid search, random search, Bayesian optimization, genetic algorithms, gradient boosting trees, automated machine learning (AutoML), etc.
Ensemble learning and final validation	Voting classifiers, stacked models, random forests, gradient boosting trees, deep learning ensembles, etc.

#### 4.1.3 Model interpretation and results analysis

After the processes of model selection, training, and tuning, it is essential to interpret the final model and its outcomes using robust methodological tools. Local interpretability analysis, for instance, employs techniques like Local Interpretable Model-Agnostic Explanations (LIME). LIME constructs interpretable surrogate models for specific samples, allowing researchers to identify key factors—such as vaccination rates, hospital accessibility, or community outreach programs—that significantly influence the classification of public health interventions as “effective” or “ineffective.” This approach facilitates a nuanced understanding of decision-making in individual cases, such as assessing the effectiveness of a vaccination campaign in a particular region.

Global interpretability analysis utilizes Shapley Additive Explanations (SHAP) values to quantify the contribution of each feature across the entire dataset. In evaluating public health policies, SHAP values provide insights into how variables such as healthcare expenditure, population density, and disease prevalence influence model predictions. For example, a SHAP summary plot might demonstrate that higher healthcare expenditure consistently correlates with improved health outcomes, whereas the impact of population density varies depending on regional infrastructure.

To complement these analyses, causal model visualization is used to illustrate the pathways and interactions between policy interventions and health outcomes. Directed acyclic graphs (DAGs) enable researchers to explicitly map the relationships between public health interventions, such as mask mandates or quarantine measures, and their effects on outcomes like infection rates or mortality. For instance, a DAG might show how increasing testing capacity reduces disease transmission through early detection and isolation, aligning the model’s predictions with epidemiological theory.

Model evaluation and validation are crucial for ensuring that interpretability results align with theoretical expectations and public health frameworks. These results are subsequently tested on an independent dataset to evaluate the model’s performance on unseen data, thereby confirming its robustness in predicting policy effectiveness across diverse settings.

**Figure 4:**
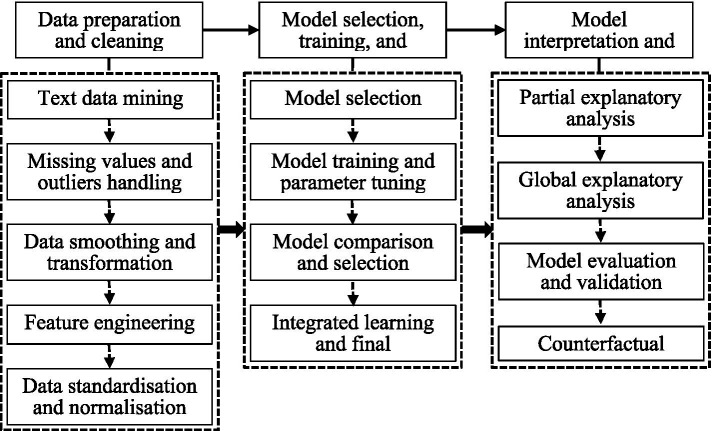
Steps in the application of machine learning to public health policy evaluation.

Counterfactual analysis is a vital component of public health policy evaluation, enabling the exploration of hypothetical scenarios. For instance, the model can simulate potential outcomes if a lockdown policy had not been implemented during a pandemic, helping researchers assess the policy’s direct and indirect effects on public health metrics such as infection rates, hospital admissions, and fatalities. This approach strengthens the model’s capacity to generate actionable insights for policymakers and public health officials. Figure 4 outlines the steps involved in applying machine learning to public health policy evaluation.

### 4.2 Application examples

Machine learning has been effectively applied in various public health policy contexts to improve evaluation accuracy and inform policy decisions. A typical example is the use of machine learning techniques to assess the effectiveness of Brazil’s smoking cessation treatment policy, which combines theory-driven public health models with data analysis. By analyzing records from 1,202 patients, key variables such as drug use, nicotine dependence level, and relapse frequency were identified. These variables were selected based on the theoretical framework of smoking cessation interventions and clinical practice, ensuring the scientific validity and interpretability of the data and model. The study utilized multiple machine learning algorithms, including support vector machines (SVM) and random forests, for training and validation, ultimately selecting SVM as the best model, with a prediction accuracy of 72.6%. Additionally, the study calculated the odds ratio (OR) for the variables to quantify their association with smoking cessation success. For instance, the importance of drug use was consistent with the theoretical model (OR = 4.42). Based on this model, Massago et al. (32) developed a user-friendly tool that predicts the probability of treatment success based on patient characteristics, helping to optimize resource allocation and personalized intervention strategies. This provides a scientifically driven, theory- and data-based foundation for public health policy implementation.

Furthermore, there are numerous other examples. For instance, Sarmiento et al. (33) highlighted the application of machine learning in COVID-19 clinical diagnosis, epidemiological variable analysis, and drug discovery through protein engineering. Kwak et al. (34) used machine learning to identify optimal COVID-19 control strategies, while Moosazadeh et al. (35) assessed the vulnerability of U.S. counties and the impact of various policies. Machine learning also plays a vital role in identifying high-risk populations, enabling targeted public health interventions. Clinical Decision Support Systems (CDSS) powered by machine learning assist clinicians in decision-making. Patel et al. (36) analyzed factors affecting maternal healthcare policies in India, providing insights for precision healthcare. Additionally, machine learning improves healthcare cost management, quality, and accessibility (37). In tobacco control, machine learning analyzes social media content to uncover factors affecting anti-smoking message dissemination and public engagement, aiding evidence-based policymaking (38, 39). Moreover, machine learning can also be applied to disease prediction and diagnosis, such as in cases of abnormal increase of transaminase in valproic acid-treated epilepsy (40) and colon cancer diagnosis and staging classification (41).

To implement machine learning in public health policy assessment, factors influencing health information systems (HIS) adoption must be addressed. Effective integration relies on system readiness to manage large-scale health data. A key determinant is the perceived ease of use and usefulness of HIS (42). Moreover, healthcare facility size and structure moderate adoption; larger hospitals possess resources and capacity for advanced technologies, whereas smaller ones face budget, expertise, and infrastructure constraints. These challenges shape machine learning adoption, especially in resource-limited settings.

## 5 Limitations and future directions of machine learning in public health policy impact assessment

In light of the data challenges presented by the era of big data, machine learning can significantly broaden the applicability of traditional public health policy effect assessments by introducing numerous new methods and application scenarios. However, this approach also has certain limitations in the context of public health policy effect evaluation, which will be examined in this section. Based on these discussions, we will propose directions for the future development of public health policy effect assessments and machine learning methodologies.

### 5.1 Limitations of machine learning in public health policy impact assessment

#### 5.1.1 Challenges in explanation brought by black-box models

As previously noted, machine learning addresses the mis-specification issues inherent in traditional public health policy effect assessment methods through its data-driven approach. However, the complexity of these models can lead to difficulties in result interpretation. Machine learning models are generally classified as either white-box or black-box models (43). White-box models, such as linear regression and single decision trees, provide relatively straightforward mappings from inputs to outputs, making them easier to understand but also more susceptible to mis-specification. In contrast, black-box models—including neural networks, support vector machines, and random forests—enhance model accuracy by capturing intricate variable relationships. Nonetheless, the use of black-box models introduces a range of new challenges. Scholars often understand only the inputs and outputs of these models, lacking insight into the specific decision-making processes, parameter settings, and feature processing that occur internally. This lack of transparency complicates the interpretation and validation of the models, potentially rendering the derived conclusions less meaningful due to their inability to be reasonably interpreted (44). Therefore, achieving a balance between data-driven approaches and theory-supported relationships to ensure model interpretability and credibility poses a significant challenge for machine learning in the realm of public health policy evaluation.

#### 5.1.2 Issues of data bias and the perpetuation of historical inequalities

As previously discussed, the advent of big data enables the application of machine learning methods that do not rely on specific models or functional forms. Instead, these methods leverage large-scale datasets to train the model, allowing the data itself to reveal the underlying functional relationships and facilitating a transition from theory-driven to data-driven assessments of public health policy effects. However, this data-driven approach can also introduce data bias into machine learning evaluations of public health policy. On one hand, systematic biases may arise during the data collection process, leading to the underestimation or overestimation of certain groups or characteristics within the training data. Such biases may stem from socioeconomic factors, limitations in technical access, or flaws in data collection methodologies (48). For instance, if certain patients lack access to healthcare resources due to poverty or the digital divide, their representation in the training data may be inadequate, resulting in their underrepresentation in public health policy assessments. This data collection bias can reflect existing societal inequalities and disparities, introducing distortions in machine learning models that hinder accurate assessments of policy effects on these groups. On the other hand, data-driven approaches may inadvertently perpetuate historical data biases that reinforce past inequalities and social injustices. If historical data contains biases or reflects unequal treatment of certain groups, machine learning models may internalize these biases, leading to unfair predictions in public health policy evaluations. For example, if a particular group has historically experienced discriminatory treatment in healthcare, the model may disproportionately emphasize the negative aspects of this group while neglecting their positive attributes, thus rendering public health policy effect assessments incomplete and unjust.

#### 5.1.3 Data privacy and ethical issues

Large-scale public health datasets contain a wealth of sensitive information, and the application of machine learning models can raise significant data privacy and ethical concerns during the processing of this data. Data privacy issues primarily arise from the presence of sensitive information within these datasets, such as individuals’ identities, health statuses, and lifestyles. When this information is utilized for public health policy evaluation, there is a potential risk of misuse, which could result in targeting individuals, discriminatory decision-making, and privacy violations. Furthermore, machine learning models may inadvertently lead to information leakage while processing high-dimensional, unstructured public health data, thereby jeopardizing individual privacy. Additionally, the potential misuse of data represents a serious ethical issue. Once public health data is collected, there is a risk that it may be used for unauthorized purposes or combined with other datasets to extract additional information. This shift in purpose can threaten individual rights and provoke privacy and ethical controversies.

### 5.2 Future directions of public health policy impact assessment and machine learning methods

#### 5.2.1 Combining data-driven and theory-driven approaches to enhance model interpretability

As noted earlier, theory-driven approaches often fail to capture the complex, nonlinear relationships in public health data, while data-driven black-box models reduce interpretability. To optimize the use of public health data and machine learning methods, future research should adopt a combined “data-driven and theory-driven” framework. Theory provides *a priori* knowledge on public health variables, guiding the construction of data-driven models. Integrating theoretical frameworks during model development allows for more targeted model selection and construction, enhancing interpretability and credibility. For example, Cao et al. (45) used a theory-driven approach to study geographic factors influencing healthcare expenditures, offering insights for policy interventions. A data-driven approach can also validate theoretical assumptions by comparing the results of theory-guided and data-driven models. This comparison can assess the alignment of theoretical frameworks with real-world data, providing a more comprehensive understanding of their applicability and limitations. Moreover, the integration of theory and machine learning fosters innovation in public health theory through predictive analysis (46). By revealing previously unrecognized patterns, machine learning can inspire new theoretical perspectives and guide future research directions.

#### 5.2.2 Developing a multi-level data strategy to mitigate data bias and perpetuation of historical inequalities

To mitigate data collection bias in big data-driven machine learning for public health policy assessment, a multilevel strategy is essential. Enhancing the quality and diversity of data collection across populations can reduce biases from socioeconomic factors, technological limitations, and flawed methods. Integrating diverse data sources, such as electronic health records, demographic surveys, and social media, improves representativeness, reducing the risk of misestimating certain groups. Bias detection tools like Fairness Indicators and IBM AI Fairness 360 can help identify disparities in data coverage and model outputs. A transparent, accountable data collection process is vital, supported by clear standards, privacy protections, and open data-sharing principles. Adopting FAIR (Findable, Accessible, Interoperable, and Reusable) principles ensures equitable data practices. Strengthened collaboration with community organizations and stakeholders further ensures comprehensive representation of diverse health needs. To address the impact of historical data on models, data balancing techniques such as SMOTE (Synthetic Minority Oversampling Technique) and reweighting can correct biases by focusing on underrepresented populations during training. Combined with continuous fairness assessments, these techniques ensure model predictions align with public health policy equity goals. The specific application steps for methods such as Fairness Indicators, IBM AI Fairness 360 (AIF360), and SMOTE are outlined in the Table 2.

**Table 2:** Steps and techniques for detecting and addressing bias and inequality.

Fairness indicators	
Defining subgroups	Identify the subgroups that need to be evaluated based on the research objectives. For instance, in public health policy evaluation, subgroups can be defined by characteristics such as gender, age, race, or region.
Data collection and preparation	Prepare datasets containing subgroup labels, ensuring the data format is compatible with the analytical tools. The dataset should also include target variables and predictions.
Selecting evaluation metrics	Decide on the metrics to assess model performance, such as accuracy, AUC, recall, etc.
Running Subgroup Analysis	The tool will split the data according to the defined subgroups and calculate metric values for each subgroup. This helps observe performance differences across various groups.
Generating visual reports	The tool provides visualizations, such as bar charts or heatmaps, to compare performance differences among subgroups. These visualizations help identify groups with the largest biases.
Interpreting results and improving the model	Based on the evaluation results, identify subgroups with performance imbalances and adjust the data, model, or algorithms to reduce biases. For instance, add more training data or redesign the model for underperforming subgroups.
IBM AI Fairness 360 (AIF360)	
Define fairness objectives	Clearly identify the fairness concerns in your research. For example, determine whether the model should provide equal opportunities for individuals of different races or genders in its predictions.
Prepare the dataset	Ensure the dataset includes sensitive attributes (e.g., gender, race) along with the target variable. AIF360 supports various data formats and provides benchmark datasets for researchers.
Select fairness metrics	Choose appropriate metrics based on the research goals, such as Demographic Parity, Equal Opportunity, or Disparate Impact.
Conduct bias detection	The tool will calculate disparities between different groups based on the selected metrics, quantifying the model’s unfairness. For example, it might reveal that the positive prediction rate for one gender is significantly lower than for others.
Choose bias mitigation methods	Address the identified biases by selecting suitable mitigation strategies, such as pre-processing, in-processing, or post-processing methods.
Reevaluate the model	Recompute fairness metrics using the adjusted data or model to determine whether the mitigation measures effectively reduced biases.
SMOTE	
Analyze class distribution	Examine the sample counts for each class in the dataset to identify imbalanced classes (e.g., a severe disparity between positive and negative samples). Determine the target class (minority class) and the number of synthetic samples required.
Select an appropriate feature space	SMOTE generates synthetic samples in the feature space of the minority class. Ensure that the selected features are relevant to the classification task. If the dataset contains noise or outliers, preprocess the data first (e.g., denoising, normalization).
Generate synthetic samples	Select Nearest Neighbors, Create New Samples, Adjust Generation Ratio
Integrate new data	Combine the synthetic samples with the original dataset to create a new training dataset. Ensure the data distribution is balanced and avoid generating excessive minority class samples, which could introduce bias.
Evaluate model performance	Train the model on the balanced dataset and compare its performance metrics (e.g., accuracy, recall, F1 score) with those before balancing. Check if the model demonstrates improved recognition of minority class samples while avoiding increased misclassification of majority class samples.

#### 5.2.3 Integrating technical, legal, and social oversight to ensure data privacy and ethical issues

Addressing data privacy and ethics in public health requires technical, legal, and social strategies. Differential privacy techniques, like those used by the U.S. Census Bureau in 2020, protect individual data by adding noise while enabling trend analysis (47). Advanced encryption, such as homomorphic encryption and secure multi-party computation frameworks like OpenMined, safeguards privacy during data processing. These methods balance privacy with analytical utility, proving effective in public health. Legal frameworks like the EU’s GDPR and U.S. HIPAA enforce data protection through consent requirements and privacy protocols. Institutions such as the UK’s Information Commissioner’s Office and independent review boards (IRBs) strengthen oversight and ethical compliance. Social engagement, through programs like the U.S. All of Us Research Program and community advisory boards, ensures accountability. Participatory workshops, citizen juries, media, and NGOs, like Privacy International, promote transparency, address privacy concerns, and advocate for ethical practices.

#### 5.2.4 Employing validation and benchmarking strategies to ensure robustness and reproducibility

To enhance the accuracy and reliability of machine learning models in public health policy evaluations, it is crucial to employ robust validation and benchmarking strategies. Validation can be achieved using standard performance metrics, including precision, recall, and the area under the receiver operating characteristic curve (AUC-ROC). Furthermore, cross-validation techniques, such as k-fold cross-validation, help assess a model’s generalizability across different data subsets. Benchmarking involves utilizing standardized datasets that reflect the scope of public health policies, enabling comparative evaluations of various machine learning approaches. For instance, public datasets like health insurance claims or national epidemiological surveys offer a consistent baseline for model performance assessment. Combining these strategies ensures the robustness of machine learning applications while promoting transparency and reproducibility in policy evaluations.

## 6 Conclusion and discussion

Current empirical research in public health primarily focuses on evaluating the effects of public health policies. Scientific policy evaluation helps decision-makers understand the real impact of policy implementation, identify potential issues and deficiencies, and provide critical support for policy adjustments and improvements. This paper summarizes the principles, applicability, and specific model settings of three traditional public health policy evaluation methods: the difference-in-differences method, regression discontinuity design, and synthetic control method. It also examines the challenges these methods face in the era of big data, including unstructured health data, large-scale data processing, and model-related issues such as misspecification, multicollinearity, and overfitting. In response, this paper explores how machine learning addresses these challenges. Machine learning can handle unstructured health data and large-scale data more effectively, while resolving limitations in traditional models. Specifically, it corrects model misspecification through data-driven approaches, mitigates multicollinearity by reducing high-dimensional models, and tackles overfitting through regularization techniques. This enhances the scope, analytical dimensions, and practical value of public health policy evaluation.

However, machine learning also introduces new challenges in public health policy evaluation, as outlined in this paper: black-box models hinder result interpretation, data bias risks reinforcing historical health inequalities, and data privacy and ethical concerns emerge. Therefore, relying solely on machine learning for public health policy evaluation is limited and requires integration with theoretical and practical insights from the public health domain. This paper proposes several strategies for advancing the use of machine learning in public health policy evaluation: combining data-driven and theory-driven approaches to enhance model interpretability, developing a multi-level data strategy to address data bias and the perpetuation of historical health inequalities, and integrating technical measures, legal frameworks, and social oversight to safeguard data privacy and ensure ethical use.

Practical integration requires developing hybrid models that combine machine learning’s data exploration strengths with established theoretical frameworks. Effective collaboration between data scientists and public health experts is crucial. For instance, jointly developing validation frameworks based on epidemiological theories can enhance the interpretability of machine learning applications. Additionally, pilot studies comparing purely data-driven and hybrid models can assess their effectiveness and feasibility across various health policy contexts. Implementing multi-level data strategies requires comprehensive data governance frameworks. Public health agencies can collaborate with community organizations to ensure diverse representation in data collection. The feasibility of real-world applications can be enhanced by leveraging federated learning, which enables data sharing across institutions while maintaining privacy, thus overcoming challenges in creating unified datasets. A practical step is forming interdisciplinary committees of legal experts, technologists, and public health practitioners to oversee the implementation of safeguards. These committees could create use-case templates for ethical machine learning applications in health policy, addressing data privacy concerns with tools like differential privacy algorithms. Public outreach initiatives can increase transparency and build trust among stakeholders.

## Data Availability

The original contributions presented in the study are included in the article/supplementary material, further inquiries can be directed to the corresponding authors.

## References

[1] Li ZJ. Accelerating the establishment of public policy evaluation system with Chinese characteristics. J Manag World. (2022) 38:84–92. doi: 10.19744/j.cnki.11-1235/f.2022.0169

[2] Tao XH Guo F. Policy effect heterogeneity evaluation and machine learning methods: research Progress and future orientation. J Manag World. (2023) 39:216–37. doi: 10.19744/j.cnki.11-1235/f.2023.0127

[3] Xiao Z Zhou B. An Econometrician's perspective on big data. Fin Minds. (2019) 4:124–44. doi: 10.20032/j.cnki.cn10-1359/f.2019.01.006

[4] Hong YM Wang SY. How is big data changing economic research paradigms? J Manag World. (2021) 37:40–56. doi: 10.19744/j.cnki.11-1235/f.2021.0153

[5] Huang NJ Yu MZ. The Progress of research on the influence of machine learning on economic research. Econ Dyn. (2018) 129:115–29.

[6] Li LB Liu BL. Prospect for major issues of China's regional economic development during the 14th five-year plan period. J Manag World. (2020) 36:36. doi: 10.19744/j.cnki.11-1235/f.2020.0068

[7] Grimmer J. We are all social scientists now: how big data, machine learning, and causal inference work together. Polit Sci Polit. (2015) 48:80–3. doi: 10.1017/S1049096514001784

[8] Varian HR. Causal inference in economics and marketing. Proc Natl Acad Sci USA. (2016) 113:7310–5. doi: 10.1073/pnas.1510479113 27382144 PMC4941501

[9] Guo R Cheng L Li J. A survey of learning causality with data: problems and methods. ACM Comput Surv. (2018) 53:1–37. doi: 10.1145/3397269

[10] Mullainathan S Spiess J. Machine learning: an applied econometric approach. J Econ Perspect. (2017) 31:87–106. doi: 10.1257/jep.31.2.87

[11] Guo F Tao XH. Machine learning and causal relationship in social science: a literature review. China Econ Q. (2023) 23:1–17. doi: 10.13821/j.cnki.ceq.2023.01.01

[12] Shen Y Li XY Zhou QK. Estimation and inference of treatment effects with panel data in the big data era. J Quant Technol Econ. (2022) 39:120–39. doi: 10.13653/j.cnki.jqte.2022.06.007

[13] Shen XL Qian QW. Data element governance dilemma and prevention mechanisms—a perspective of value and risk integration. J Inform Resour Manag. (2023) 13:17. doi: 10.13365/j.jirm.2023.06.017

[14] Card D Krueger AB. Minimum wages and employment: a case study of the fast-food industry in New Jersey and Pennsylvania. Am Econ Rev. (2000) 90:1397–420.

[15] Guo JC Zheng WW Qu X. Policy evaluation methods, strategies and China advantages of multi-dimensional spillover effects: perspective of spatial network econometric models. Bull Natl Nat Sci Found China. (2023) 37:953–62. doi: 10.16262/j.cnki.1000-8217.2023.06.008

[16] Thistlethwaite DL Campbell DT. Regression-discontinuity analysis: an alternative to the ex-post facto experiment. J Educ Psychol. (1960) 61:309–17. doi: 10.1037/h0044319

[17] Hahn J Todd P van der Klaauw W. Identification and estimation of treatment effects with a regression-discontinuity design. Econometrica. (2008) 69:201–9. doi: 10.1111/1468-0262.00183

[18] Lee DS Lemieux T. Regression discontinuity designs in economics. J Econ Lit. (2010) 48:281–355. doi: 10.3386/w14723

[19] Hong YM Wang SY. Big data, machine learning, and statistics: challenges and opportunities. Chin J Comput. (2021) 1:17–35.

[20] Hu Y Chen HQ Qi YF. The summary of the 2nd China forum for scholars on econometrics. Econ Res J. (2019) 54:199–203. doi: 10.1360/j.cnki.er.2019.03.002

[21] Yang DF Yun Z. Innovative research on public policy evaluation in the big data era: a process-based perspective. E-Gov. (2020) 2:92–9. doi: 10.16582/j.cnki.dzzw.2020.02.010

[22] Xu ZB Feng ZY Guo XH. Frontier topics in management and decision-making driven by big data. J Manag World. (2014) 11:158–63. doi: 10.19744/j.cnki.11-1235/f.2014.11.015

[23] Wang YZ Jin XL Cheng XQ. Network big data: present and future. Chin J Comput. (2013) 36:1125–38. doi: 10.3724/SP.J.1016.2013.01125, PMID: 37113526

[24] Zhang T Li JC. Network infrastructure, inclusive green growth, and regional inequality: from causal inference based on double machine learning. J Quant Technol Econ. (2023) 40:113–35. doi: 10.13653/j.cnki.jqte.20230310.005

[25] Huang LW Jiang BT Lv SY. A survey of recommendation systems based on deep learning. Chin J Comput. (2018) 41:1619–47.

[26] Tu LC. The philosophical narration of deep learning and its essence. Acad Exch. (2022) 11:27–91.

[27] Athey S Imbens GW. Machine learning methods for estimating heterogeneous causal effects. Stat. (2015) 1050:234–43. doi: 10.1073/pnas.1510489113

[28] Athey S Imbens GW. Recursive partitioning for heterogeneous causal effects. Proc Natl Acad Sci USA. (2016) 113:7353–60. doi: 10.1073/pnas.1518407113 27382149 PMC4941430

[29] Shu XH Liu JP. Issues in handling multicollinearity using principal component regression. Stat Decis. (2004) 10:25–6.

[30] Li JH Guo YH. Principal component evaluation—a multivariate evaluation method expanded from principal component analysis. J Ind Eng Eng Manag. (2002) 1:39–46.

[31] Zhang Y Liu JW Zuo X. Survey of multi-task learning. Chin J Comput. (2020) 43:1340–78.

[32] Massago M Massago M Iora PH. Applicability of machine learning algorithm to predict the therapeutic intervention success in Brazilian smokers. PLoS One. (2024) 19:e0295970. doi: 10.1371/journal.pone.0295970 38437221 PMC10911606

[33] Sarmiento Varón L González-Puelma J Medina-Ortiz D. The role of machine learning in health policies during the COVID-19 pandemic and in long COVID management. Front Public Health. (2023) 11:1140353. doi: 10.3389/fpubh.2023.1140353 37113165 PMC10126380

[34] Kwak GH Ling L Hui P. Deep reinforcement learning approaches for global public health strategies for COVID-19 pandemic. PLoS One. (2021) 16:e0251550. doi: 10.1371/journal.pone.0251550 33984043 PMC8118301

[35] Moosazadeh M Ifaei P Tayerani Charmchi AS. A machine learning-driven Spatio-temporal vulnerability appraisal based on socio-economic data for COVID-19 impact prevention in the U.S. counties. Sustain Cities Soc. (2022) 83:103990. doi: 10.1016/j.scs.2022.103990 35692599 PMC9167466

[36] Patel SS. Explainable machine learning models to analyse maternal health. Data Knowl Eng. (2023) 146:102198. doi: 10.1016/j.datak.2023.102198, PMID: 39790414

[37] Ball HC. Improving healthcare cost, quality, and access through artificial intelligence and machine learning applications. J Healthc Manag. (2021) 66:271–9. doi: 10.1097/JHM-D-21-00149 34228686

[38] Lin SY Cheng X Zhang J Yannam J Barnes A Koch J. Social media data Mining of Antitobacco Campaign Messages: machine learning analysis of Facebook posts. J Med Internet Res. (2023) 25:e42863. doi: 10.2196/42863, PMID: 36780224 PMC9972210

[39] Elmitwalli S Mehegan J Wellock G Gallagher A Gilmore A. Topic prediction for tobacco control based on COP9 tweets using machine learning techniques. PLoS One. (2024) 19:e0298298. doi: 10.1371/journal.pone.0298298, PMID: 38358979 PMC10868820

[40] Ma H Huang S Li F Pang Z Luo J Sun D. Development and validation of an automatic machine learning model to predict abnormal increase of transaminase in Valproic acid-treated epilepsy. Arch Toxicol. (2024) 98:3049–61. doi: 10.1007/s00204-024-03803-5, PMID: 38879852

[41] Su Y Tian X Gao R Guo W Chen C Chen C. Colon Cancer diagnosis and staging classification based on machine learning and bioinformatics analysis. Comput Biol Med. (2022) 145:105409. doi: 10.1016/j.compbiomed.2022.105409, PMID: 35339846

[42] Luo J Ahmad SF Alyaemeni A Ou Y Irshad M Alyafi-Alzahri R. Role of perceived ease of use, usefulness, and financial strength on the adoption of health information systems: the moderating role of hospital size. Humanit Soc Sci Commun. (2024) 11:1–12. doi: 10.1057/s41599-024-02976-9, PMID: 39310270

[43] Li B Wang WX. Improvement of classification model by NCA dimension reduction and Bayesian optimization parameter adjustment. Comput Appl Softw. (2019) 36:281–99.

[44] Li XW Shu H Guang Y. Survey of the application of natural language processing for resume analysis. Comput Sci. (2022) 49:66–73.

[45] Cao P Pan J. Understanding factors influencing geographic variation in healthcare expenditures: a small areas analysis study. Inquiry. (2024) 61:469580231224823. doi: 10.1177/00469580231224823 38281114 PMC10823849

[46] Chen YS Wu XG Hu AN. Social prediction: a new research paradigm based on machine learning. Soc Stud. (2020) 35:94–244. doi: 10.19934/j.cnki.shxyj.2020.03.005

[47] Yang SL. Personal information privacy protection method based on explicit and implicit feedback. Inf Sci. (2023) 41:134–40. doi: 10.13833/j.issn.1007-7634.2023.11.016

[48] Ribeiro MT Singh S Guestrin C. “Why should I trust You?”: Explaining the predictions of any classifier. Proc. 22nd ACM SIGKDD. Int. Conf. Knowledge Discov. Data Min. San Francisco, California, USA: ACM, (2016). 1135–1144. doi: 10.48550/arXiv.1602.04938

